# IL-17 cytokines in bone healing of diabetic Charcot arthropathy patients: a prospective 2 year follow-up study

**DOI:** 10.1186/s13047-015-0096-3

**Published:** 2015-08-18

**Authors:** Agnetha Folestad, Martin Ålund, Susanne Asteberg, Jesper Fowelin, Ylva Aurell, Jan Göthlin, Jean Cassuto

**Affiliations:** Department of Orthopaedics, CapioLundby Hospital, Göteborg, Sweden; Department of Orthopaedics, Sahlgrenska University Hospital, Mölndal, Sweden; Diabetes Care Unit, Department of Medicine, Frölunda Specialist Hospital, Västra Frölunda, Göteborg, Sweden; Department of Radiology, Sahlgrenska University Hospital, Mölndal, Sweden; Orthopaedic Research Unit, Sahlgrenska University Hospital, Staben, Hus U1, 431 80 Mölndal, Sweden, Göteborg University, Göteborg, Sweden

**Keywords:** Charcot arthropathy, Bone healing, Offloading, Interleukin-17, Diabetes

## Abstract

**Background:**

Little is currently known of the pathophysiological mechanisms triggering Charcot arthropathy and regulating its recovery although foot trauma has been proposed as a major initiating factor by activation of proinflammatory cytokines leading to increased osteoclastogenic activity and progressive bone destruction. Several members of the IL-17 family of proinflammatory cytokines have been shown to play a key role in the pathogenesis of inflammatory conditions affecting bone and joints but none has previously been studied in Charcot foot patients. The aim of this study was to investigate the role of IL-17A, IL-17E and IL-17F in patients presenting with Charcot foot.

**Methods:**

Twenty-six consecutive Charcot patients were monitored during 2 years by repeated foot radiographs, MRI and circulating levels of IL-17A, IL-17E and IL-17F. Analysis of cytokines was done by ultra-sensitive chemiluminescence technique and data were analyzed by one-way repeated measures ANOVA. Neuropathic diabetic patients (n = 20) and healthy subjects (n = 20) served as controls.

**Results:**

Plasma IL-17A and IL-17E in weight-bearing Charcot patients at diagnosis were at the level of diabetic controls, whereas IL-17F was significantly lower than diabetic controls. A significant increase in IL-17A and IL-17E reaching a peak 2–4 months after inclusion and start of offloading treatment in Charcot patients was followed by a gradual decrease to the level of diabetic controls at 2 years postinclusion. In contrast, IL-17F increased gradually from inclusion to a level not significantly different from diabetic controls after 2 years.

**Conclusions:**

Charcot patients display a significant elevation of all three IL-17 cytokines during the follow-up period relative values at diagnosis and values in control patients supporting a role in the bone repair and remodeling activity during the recovery phase. The rapid increase of IL-17A and IL-17E shortly after initiating off-loading treatment could suggest this to be a response to immobilization and stabilization of the diseased foot.

## Background

The Charcot foot or Charcot arthropathy is a complex and potentially limb-threatening, destructive process often recognized by early signs such as pain, warmth, edema, or pathologic fractures in a neuropathic foot [1]. We currently know little of the pathophysiology of Charcot other than that it involves multiple background factors such as peripheral neuropathy, with its secondary effects on circulation, sensory input and foot biomechanics, and in most cases diabetes. The triggering mechanism(s) remain however elusive although trauma has been proposed as one such factor. The conjunction of these background factors has been proposed to explain the association of features that are typical of this disorder, namely osteolysis and vascular calcification in association with intact lower limb blood flow [2]. However, the acute Charcot foot is also characterized by a pronounced inflammatory reaction, the pathogenic significance of which has been the focus of several review articles in recent years [2, 3]. In an early review [4], the author proposed that an initial trauma could set off an inflammatory cascade resulting in increased osteoclastogenesis due to disturbance in the balance between RANKL and OPG. The role of the RANKL/OPG-pathway was investigated in a recent study showing that at diagnosis and onward, this pathway is primarily involved in bone remodeling whereas the powerful bone anabolic Wnt/β-catenin pathway, which had not been previously investigated in Charcot arthropathy patients, plays a critical role in remodeling as well as preservation of the foot skeleton in the acute and chronic phases of the disease [5].

Despite decade-long dominant role assigned to the proinflammatory cytokines belonging to the T-helper 1 and T-helper 2 cell (Th1/Th2) subsets of the immune system (e.g. IL-6, IL-1β and TNF-α) as the driving force for the development and maintenance of inflammation-driven bone- and joint diseases [6], it is now evident that Th1/Th2 cannot alone account for the pathogenesis of these disorders [7] and that many inflammatory conditions are partly or largely driven by a new subset, namely the T-helper 17 (Th17) cells [8], which are the main source of IL-17 cytokines. This family is composed of six members ranging from IL-17A to IL-17F which exert their biological actions through activation of several cascade systems, such as NFκB and kinase signaling pathways [9]. A growing number of studies in animals and humans have shown IL-17A to be pivotal in a multitude of bone-destructive processes, either by directly or indirectly stimulating osteoclastogenesis [10] or by synergizing with other proinflammatory cytokines, such as TNF-α and IL-1β, in their bone destructive role [10]. Recent studies have shown that, despite being related, these cytokines have differential, non-overlapping and sometimes opposing effects [11, 12]. Despite Charcot arthropathy being a bone degenerative condition, we are not aware of any study investigating the role of this important family of proinflammatory cytokines in Charcot patients.

This longitudinal observational study was designed to explore the role of IL-17A, IL-17E and IL-17F in the pathophysiology of Charcot arthropathy by measuring plasma levels of the cytokines at presentation and repeatedly during 2 years until full mineralization of the foot skeleton and no inflammatory signs occurred on radiographs.

## Methods

This observational prospective study was approved 2007 by the Review Board of Västra Götalands Regionen (EPN D-number 499–07) with additional approval 2010 (T 762–10 ad 499–07). All the subjects provided informed consent before participating in the study. The study complies with the STROBE-statement for observational studies [13]. We tested the following hypotheses: (1) do plasma levels of IL-17 cytokines differ between healthy subjects and diabetic patients with documented peripheral neuropathy (2) are plasma levels of IL-17 cytokines in Charcot patients at presentation and 2 years postinclusion different from diabetic control patients or healthy subjects and (3) do the levels of IL-17 cytokines change over time in Charcot patients and could such changes be related to off-loading treatment.

### Patient selection and treatment

Twenty-eight consecutive ambulatory men and women admitted to Sahlgrenska University Hospital with clinical signs of unilateral acute Charcot’s foot were included in the study during 2009–2014. Two patients deceased shortly after inclusion and were excluded. One patient interrupted participation 19 months into the study after being informed that radiographs showed finalized bone healing, but did not object to data being processed in the study. Inclusion of Charcot patients was based on medical history, clinical examination and radiological findings. Patients were included in the Charcot group if they met the following criteria: (1) Type 1 or 2 diabetes with duration of ≥ 1 year (2) peripheral bilateral neuropathy as defined below and (3) clinical signs of active Charcot arthropathy with hot/reddened/swollen foot and skin temperature in the affected foot of ≥ 2 °C higher than in the contralateral foot. Within a day of inclusion, Charcot patients received a non-weight bearing total contact cast (TCC). The cast was repeatedly replaced as required by changes in foot volume due to increased or attenuated swelling. The non-weight-bearing protocol was aided by crutches or wheel-chair and was continued until the difference in skin temperature between the feet was ≤ 1 °C and no signs of redness and swelling were present for the past 30 days. TCC was then replaced by orthosis for partial weight-bearing. Evaluation of skin temperature and toe pressure continued until 2 years postinclusion. When full weight-bearing was allowed, the patient received prescribed accommodative shoes. Bilateral foot radiograhs and magnetic resonance imaging (MRI) at inclusion and repeatedly during the follow-up period were used to monitor dislocations, fractures and soft tissue- or bone marrow edema. Exclusion criteria were plantar ulcerations, documented history of trauma or surgery involving bone tissue during the past year prior to inclusion, current immunosuppressive therapy (steroids, cancer treatment) or medication known to affect bone metabolism (e.g. bisphosphonates, denosumab). A control group of twenty ambulatory neuropathic patients with diabetes type 1 or 2 was included at Frölunda Specialist Hospital during 2011 and another group of twenty healthy subjects was included at Sahlgrenska University Hospital/Mölndal during 2010. Both control groups lacked documented history of joint/bone disease or bone trauma/surgery one year prior to inclusion and received no osteoporosis medication.

### Skin sensitivity, skin temperature and toe pressure

Skin sensitivity was measured in Charcot patients and diabetic control patients bilaterally at inclusion on 4 different locations of the foot using the Semmes-Weinstein monofilament test [14]. The monofilament (10 g) was pressed against the skin and the patient’s ability or inability to feel the sensation upon buckling of the monofilament was registered. Neuropathy was present if three or more sites were insensate to the monofilament. Skin temperature was measured bilaterally on the dorsal foot 5 cm distal of the ankle and 2 cm proximal of the mid toe. Toe blood pressure was measured bilaterally using a specially designed cuff [15]. Measurements of skin temperature and toe pressure were performed at inclusion, 1 week, 2, 4, 6, 8, 12, 18 and 24 months thereafter.

### Blood samples

Peripheral venous blood was collected for routine analyses (Hemoglobin, serum creatinine, CRP, SR and HbA1c) and for cytokine analyses (pre-chilled EDTA tubes immediately centrifuged at 3000xg for 10 min at 4 °C before being stored at −85 °C until analysis). Blood was collected in the Charcot group at inclusion, 1 week, 2, 4, 6, 8, 12, 18 and 24 months after inclusion. In the diabetic control group and the group of healthy subjects, blood was sampled on a single occasion and the biomarker values obtained were used for comparisons with Charcot patients both at inclusion and at 2 years postinclusion. Before analysis, samples were thawed on ice before being mounted on assay plates and analyzed for IL-17A, IL-17F, and IL-17E.

### Determination of plasma biomarkers by ECL technology

The Sector Imager 2400 assay platform from MesoScaleDiagnostics (MSD, Gaithersburg, USA) was used for biomarkers analysis (for details see www.mesoscale.com). This electrogenerated chemiluminescence technique (ECL) is a high-sensitivity assay platform with low detection limit and wide dynamic range compared to colorimetric ELISAs [16]. ECL uses plates with integrated screen-printed carbon ink electrodes on the bottom of the wells (a working electrode and a counter electrode generating an electrical circuit) which act as both the capture phase for solid phase immunoassays and the source of electrochemical energy for inducing chemiluminescence. The capture antibody is attached to the carbon bottom surface of the well and forms a sandwich with a biotinylated detection antibody encapsulating the targeted plasma protein. MSD Sulfo-TAG™ reagent is added and forms a strong bond to the detection antibody by biotin-streptavidin interactions. The ruthenium-based tag is used in combination with an enhancer, tripropylamine. The tag undergoes rapid redox-reaction that emits light at 620 nm when excited by an electrical impulse applied to the carbon electrode. Photon levels from the Sulfo-TAG™ are imaged by a cooled (−28 °C) CCD camera housed in the Sector Imager plate reader. Only tag bound antibody-protein complexes in proximity of the electrode are detected. Photon readings from each plate are transferred to excel-based pivot table and data for each cytokine is expressed against known standards.

Quantitative measurements were done by use of a matched pair of anti-human (ah) antibodies i.e. capture antibody (CA) and biotinylated detection antibody (DA). Standard curves were created by use of the corresponding human recombinant proteins (hRP). Samples were mounted on uncoated standard plates from MSD (L15XA). IL-17A:CA/DA/hRP (Peprotech cat no.900-K84), IL-17E:CA/DA/hRP (Peprotech cat no.900-K234) and IL-17F:CA/DA/hRP (Peprotech cat no.900-K277). Antibodies were optimized by checkerboard titrations and subsequent control of standard curves. Plasma from Charcot, diabetic controls and healthy subjects were mounted on the same plate to minimize inter-group variability. Inter-assay variations were <5 %.

### Radiography

Radiographs of both feet were performed in supine position with dorso-plantar, oblique and lateral projections as well as weight-bearing in frontal and lateral projections. MRI was performed on a 1.5 T Siemens Magnetom Symphony scanner with patients supine and feet first into the gantry. All examinations were performed by head-neck surface coil with feet in flexed position. The feet were examined with 4 sequences each without intravenous contrast medium: T1, T2, T2 3d and STIR sequences in sagittal, transverse and coronary positions. Following administration of intravenous contrast medium, transverse and sagittal projections were obtained by T-sequences. Radiographic and MRI examinations were performed at a preset schedule within a week after assigning the patient to the study and 6, 12, 18 and 24 months after inclusion.

For anatomical classification of Charcot we used the system described by Sanders and Frykberg with five different patterns depending on the areas of the foot affected [17], i.e. Pattern I: metatarsophalangeal and interphalangeal joints; Pattern II: tarso-metatarsal joints; Pattern III: naviculocuneiform, talonavicular and calcaneocuboid joints; pattern IV: ankle and subtalar joints; Pattern V: calcaneus (Table 1). A modified Eichenhotz staging based on plain X-rays as described by Sella and Barrette [18], was used for disease characteristics and comprises 5 stages: Stage 0 (warm, reddened, swollen foot and normal radiographs), stage 1 (clinical findings and radiographic cysts, erosions, localized osteopenia and occasionally diastases), stage 2 (joint subluxation), stage 3 (dislocation and arch collapse), stage 4 (healed stage of bony process) (Table 1). Inflammation in the soft-tissue and bony structures of the foot (edema), were identified by MRI and summarized in Table 2.

**Table 1:** Clinical and demographic data in Charcot patients, diabetic controls and healthy subjects

	Charcot arthropathy Inclusion	Charcot arthropathy End of study	Diabetic controls Inclusion	Diabetic controls +2Y	Healthy subjects
	(n = 26)	(n = 26)	(n = 20)	(n = 20)	(n = 20)
Age (median- range, years)	61 (42–87)	63 (42–87)	55 (20–94)	57 (20–94)	58 (24–78)
Gender (n)					
Females	10	10	8	8	9
Males	16	16	12	12	11
Diabetes type 1/2 (n)	11/15	11/15	6/14	6/14	-
Diabetes duration (years)	25 ± 4	-	14 ± 6	-	-
Debut foot symptoms (weeks, median-range)	8.4 (1–24)	-	-	-	-
Systolic blood pressure (mmHg)	149 ± 6	148 ± 6	127 ± 4	-	138 ± 4
Diastolic blood pressure (mmHg)	80 ± 3	80 ± 3	76 ± 2	-	81 ± 3
Arterial toe pressure (mmHg)					
Charcot foot	114 ± 11	122 ± 8	115 ± 4	-	-
Contralateral foot	129 ± 8	119 ± 7	114 ± 5	-	-
Skin temperature (°C)					
Charcot foot	31.3 ± 0.8^a^	29.7 ± 0.7	30,0 ± 0,4	-	-
Contralateral foot	28.8 ± 0.9	29.3 ± 0.7	30,2 ± 0,4	-	-
Pain at rest (n)	4	0	0	-	-
Pain at weight bearing (n)	17	0	0	-	-
Foot distorsion (n)	4	4	0	-	0
Total contact cast (months)	-	7.7 ± 1.0	-	-	-
Charcot pattern I/II/III/IV/V (n)	2/16/5/0/3	-	-	-	-
Nephropathy/Cardiac disease (n)	7/4	7/4	2/1	2/1	0/0
Hb (g/L)	127 ± 3	129 ± 3	136 ± 2	139 ± 3	143 ± 5
S-CRP (mg/L)	10.6 ± 3^b,c^	10.0 ± 2^d,e^	3.6 ± 0,8	3.8 ± 0,8	2.7 ± 0,7
SR (mm)	28.8 ± 6^f,g^	23 ± 5	11 ± 2	10 ± 2	6 ± 4
S-Kreatinin (μmol/L)	112 ± 19	101 ± 12	83 ± 8	89 ± 7	78 ± 5
HbA1c (mmol/mol)	60 ± 3	64 ± 2	58 ± 3	56 ± 2	-
HbA1c (%)	7.8 ± 0.3	7.4 ± 0.3	7.5 ± 0.2	7.3 ± 0.2	-

Mean ± SEM when not given differently. ^a^ *p* = 0.010 Charcot foot versus contralateral foot at inclusion. ^b^ *p* = 0.044 for Charcot at inclusion versus diabetic controls at inclusion.^c^ *p* = 0.012 for Charcot at inclusion versus healthy. ^d^ *p* = 0.038 Charcot end of study vs diabetic controls +2y. ^e^ *p* = 0.022 Charcot end of study vs healthy. ^f^ *p* = 0.001 Charcot at inclusion versus diabetic controls at inclusion. ^g^ *p* < 0.001 for Charcot at inclusion versus healthy. All other differences are not significant. Charcot pattern I-V on radiographs as described by Sanders and Frykberg (see ref: Papanas and Maltezos, 2013 [17])

**Table 2:** Staging of radiographic changes in the Charcot arthropathy foot as described by Sella and Barrette (1999) [18] based on weight-bearing radiographic examinations of the diseased foot and inflammation/bone edema on MRI. Numbers in table are number of patients

Radiographs	Inclusion	1 W	6 M	12 M	18 M	24 M
Stage 0	4	3	0	0	0	0
Stage 1	3	3	2	0	0	0
Stage 2	9	9	9	6	4	0
Stage 3	10	10	10	5	5	0
Stage 4	0	1	5	15	17	25
MRI	Inclusion	1 W	6 M	12 M	18 M	24 M
Soft tissue edema	22	22	18	14	7	0
Bone marrow edema	18	18	15	13	5	0

### Statistical methods

Repeated measures ANOVA is also referred to as within-subjects ANOVA or ANOVA for correlated samples and is a powerful tool when investigating changes in mean scores over three or more time points as was done in the Charcot group or when comparing three or more groups of patients (Charcot, diabetic controls and healthy). An important point with the current statistical design is that the same populations are being measured more than once on the same dependent variable, hence the name “repeated measures analysis”. The one-way repeated measures ANOVA used in the current study require one *dependent variable*, often referred to as an *outcome variable*, and one *independent variable* which is the variable being treated and as a result affects the dependent variable. The dependent variable needs to be *continuous* (interval or ratio) and the independent variable categorical and could be divided into categories, also called *levels*. When we repeated measurements of IL-17 in Charcot patients along the time axis, the independent variable is *time* and each level is a specific time point. The dependent variable is plasma IL-17. Thus, the main aim of the study was to examine in Charcot foot patients whether the independent variable, i.e. time from inclusion (acute phase) to study termination (chronic state), resulted in changes of the dependent variable, plasma IL-17 and whether these changes occurred in connection with TCC treatment. We were also interested knowing if there was a difference in IL-17 between Charcot patients at inclusion and study termination relative the control groups. The independent variable constituted here of individuals in the 3 groups (three levels) and the dependent variable was plasma IL-17.

Before analyzing the data by repeated measures ANOVA we tested for a number of assumptions to be met. The tested variables (biomarkers) were measured at the continuous level over time and a comparison was made between the 3 independent study groups for each individual biomarker at inclusion and at 2 years postinclusion by means of one-way repeated measures ANOVA followed by the Holm-Ŝidak test. When applicable, this model was used for comparisons between the 3 study groups for blood pressure, Hb, CRP, SR, S-kreatinin (Table 1). This statistical model ensures that the probability of incorrectly rejecting the null hypothesis for any of the pairwise comparisons in the family does not exceed alpha, thereby controlling the familywise error rate (FWE) and setting the alpha value according to Ŝidak correction of Bonferroni inequality. This stepwise method is generally more powerful than the corresponding single-step procedures. The Holm-Ŝidak test applies an accept/reject criterion on a sorted set of null hypothesis, starting from the lower *p*-value and going up to the acceptance of null hypothesis. For each comparison, the alpha value is set according to Ŝidak correction of Bonferroni inequality. The Ŝidak formula is: Ŝidak *corrected alpha* = 1 - (1 - α)^1/*k*^ where *k* (=3 in our study) is the number of comparisons performed for each individual biomarker. The Ŝidak procedure has slightly more power than the Bonferroni procedure when alpha = .05. A multiplicative model was assumed more accurate for the current data and therefore the logarithms of measurements have been used for the comparisons. Comparisons between 2 datasets of individual biomarkers within the Charcot group (e.g. inclusion vs 4 months) were performed by Wilcoxon rank sum test. Comparison of HbA1c between the Charcot and diabetic control groups was done by Mann-Whitney U test (Table 1).

As plasma IL-17 cytokines have not been previously reported in Charcot patients, we were unable to assume means and standard deviations as known quantities and could therefore not conduct an *a priori* power analysis using information from studies in the literature. A *post hoc* analysis of sample size for ANOVA based on obtained measurements of plasma IL-17 was conducted yielding a sample size of 17 when comparing 3 groups with a difference in means (20), mean SD (90), power (0.8) and alpha (0.05).

## Results

### IL-17A

**Fig. 1:**
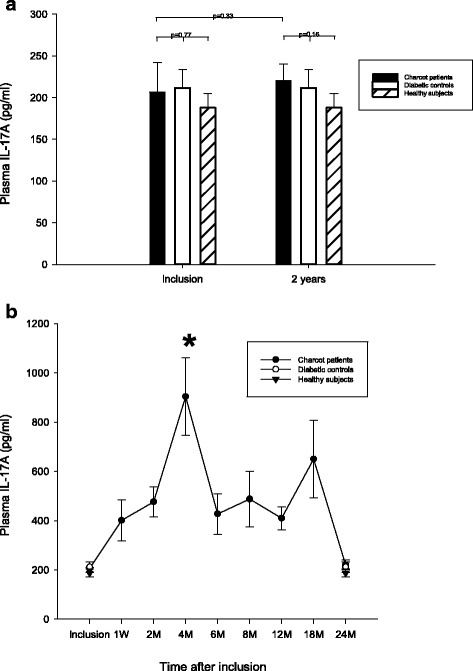
Plasma levels of IL-17A (pg/ml) in Charcot patients (n = 26), diabetes control patients (n = 20) and healthy donors (n = 20). IL-17A was not significantly different between Charcot patients, diabetic control patients and healthy subjects at inclusion into the study (*p* = 0.77) or at 2 years (*p* = 0.16). Plasma IL-17A in Charcot patients had not changed significantly at 2 years relative value at inclusion (*p* = 0.33) (1**a**). IL-17A in Charcot patients had increased significantly at 4 months relative value at inclusion (*p* = 0.002) (1**b**). Mean ± SEM

Plasma IL-17A (pg/ml) did not differ significantly between Charcot patients, diabetic controls or healthy subjects at inclusion (*p* = 0.77) or at 2 years postinclusion (*p* = 0.16) (Fig. 1a). Plasma IL-17A in Charcot patients had not changed significantly at 2 years versus value at inclusion (*p* = 0.33) (Fig. 1a). IL-17A in Charcot patients was significantly higher at 4 months versus Charcot at presentation (*p* = 0.002) (Fig. 1b).

### IL-17E

**Fig. 2:**
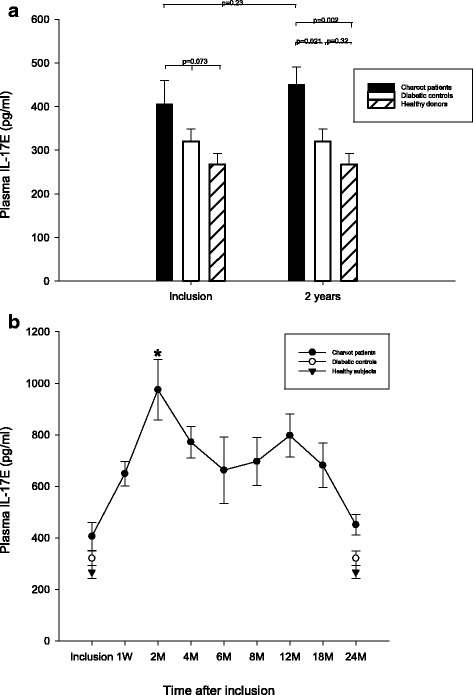
Plasma levels of IL-IL-17E (pg/ml) in Charcot patients (n = 26), diabetes control patients (n = 20) and healthy donors (n = 20). IL-17E was not significantly different between Charcot patients, diabetic control patients and healthy subjects at inclusion into the study (*p* = 0.73) (2**a**). At 2 years, IL-17E was significantly higher for Charcot patients relative diabetic controls (*p* = 0.021) and relative healthy (*p* = 0.002) but not between diabetic controls and healthy (*p* = 0.32). Plasma IL-17E in Charcot patients had not changed significantly at 2 years relative value at inclusion (*p* = 0.23) (2**a**). IL-17E in Charcot patients had increased significantly at 4 months relative value at inclusion (*p* < 0.001) (2**b**). Mean ± SEM

Plasma IL-17E at inclusion did not differ significantly between Charcot, diabetic controls and healthy subjects (*p* = 0.073) (Fig. 2a). Two years postinclusion, IL-17E was significantly higher in Charcot patients versus diabetic controls (*p* = 0.021) and versus healthy subjects (*p* = 0.002) but did not differ significantly between diabetic controls and healthy (*p* = 0.32) (Fig. 2a). Plasma IL-17E in Charcot patients at 2 years had not changed significantly from value at inclusion (*p* = 0.23) (Fig. 2a). IL-17E in Charcot patients was significantly higher at 4 months versus Charcot at presentation (*p* < 0.001) (Fig. 2b).

### IL-17F

**Fig. 3:**
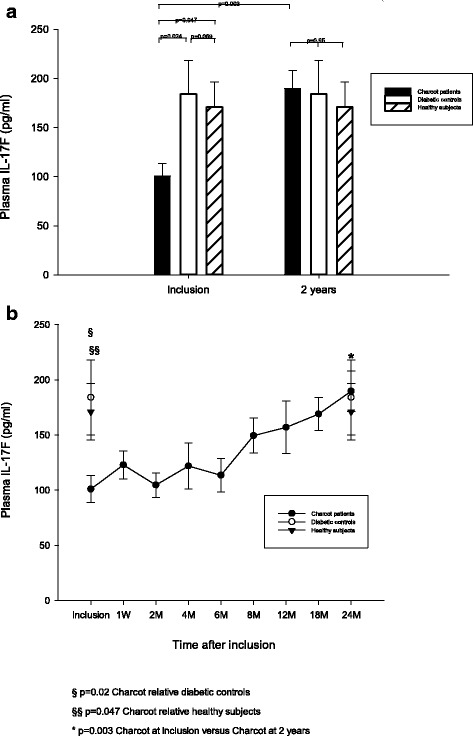
Plasma levels of IL-17F (pg/ml) in Charcot patients (n = 26), diabetes control patients (n = 20) and healthy donors (n = 20). Plasma IL-17F at start of study was significantly lower in Charcot patients versus diabetic controls (*p* = 0.024) and healthy subjects (*p* = 0.047) but not between diabetic controls and healthy (*p* = 0.69). IL-17F was not significantly different between Charcot patients, diabetic control patients and healthy subjects at 2 years (*p* = 0.95) (3**a**). IL-17F in Charcot patients was significantly higher at 2 years versus inclusion value (*p* = 0.003) (3**a**) but not at 4 months versus inclusion value (3**b**) (*p* = 0.57). Mean ± SEM

Plasma IL-17F at inclusion was significantly lower in Charcot patients relative diabetic controls (*p* = 0.024) and relative healthy subjects (*p* = 0.047), whereas difference between diabetic controls and healthy was not significant (*p* = 0.69) (Fig. 3a). Differences between Charcot patients, diabetic controls and healthy subjects at 2 years postinclusion were not significant (*p* = 0.95) (Fig. 3a). Plasma IL-17F in Charcot patients had increased significantly at 2 years relative value at inclusion (*p* = 0.003) (Fig. 3a). IL-17F in Charcot patients was not significantly higher at 4 months versus inclusion value (*p* = 0.57) (Fig. 3b).

### Radiographic data

The radiographic data are presented in table 2. One patient lacked data at 24 months due to interrupted participation. All study patients had reached complete mineralization on radiographs and no signs of inflammation on MRI at 2 years postinclusion.

## Discussion

Our results showed no significant differences between neuropathic diabetic control patients and healthy subjects for IL-17A (Fig. 1a). These results are in line with previous studies showing no significant difference in serum levels of IL-17A between type 2 diabetic patients and healthy subjects [19–21] whereas one study showed significantly lower serum level of IL-17A in neuropathic type 2 diabetic patients versus healthy subjects [22]. We are however not aware of previous studies reporting on the systemic levels of IL-17E and IL-17F in diabetic patients except for one study reporting on IL-17F in infected diabetic patients [23].

Following an immunological insult, e.g. tissue trauma, naïve T cells differentiate to mature pathogenic Th17 cells and release IL-17A, an early initiator of inflammation, which stimulates other immune cells to release proinflammatory cytokines, such as IL-1β, and chemokines thereby creating a positive autocrine feedback loop amplifying the inflammatory reaction and promoting osteoclastogenesis [24–26]. The current results showing IL-17A in Charcot patients not to differ significantly from diabetic controls at inclusion (Fig. 1) suggest that this positive feedback loop may have been inhibited [26]. In contrast to IL-17A, plasma IL-17F was significantly lower in Charcot versus diabetic controls and healthy subjects (Fig. 3). The latter finding was surprising considering the strong structural resemblance between IL-17A and IL-17F and that both are regulated by similar molecular mechanisms [11] which should make them function redundantly in promoting inflammation and bone erosion. Although both IL-17A and IL-17F induce neutrophil recruitment, recent data have shown the two cytokines to have differential effects and potencies and even to oppose each other [11, 12]. While T-helper cells are important promoters of osteolysis, they are also a source of anti-inflammatory mediators, such as IL-4, IL-10 and IL-13, being important inhibitors of osteoclastic activity. In a study by Yang et al. [11], IL-17A deficiency was shown to greatly reduce anti-inflammatory Th2 cytokines (IL-4, IL-5 and IL-13), whereas IL-17F deficiency contrarily enhanced these anti-inflammatory cytokines known to negatively regulate IL-17A [27]. This seemingly paradoxical effect by the two cytokines could help explain our findings at the time of Charcot diagnosis when the patient had exerted full weight bearing on the diseased foot. The suppression of IL-17F could thus serve as a protective measure by containing the IL-17A-induced production of osteoclastogenic pro-inflammatory cytokines and at the same time increasing the levels of anti-inflammatory cytokines.

Within a week after the start of offloading treatment, systemic levels of IL-17A started to increase more than 4-fould peaking at 4 months before gradually receding towards the end of the study (Fig. 1). This increase appears paradoxical in view of IL-17A being a strong promoter of osteoclastogenesis [24–26] and in view of radiological data showing offloading treatment to set-off the process of bone mineralization (Table 2). Similar to IL-17A, levels of IL-17E (IL-25) started to increase shortly after offloading and reached a peak after 2 month before gradually returning towards inclusion levels (Fig. 2). The striking similarity in the response by IL-17A and IL-17E came as a surprise considering that the latter cytokine belongs to a different subset of T-helper cells (Th2) than IL-17A (Th17). The main features of IL-17E, besides eosinophilia, is to inhibit the production of pro-inflammatory cytokines, such as TNF-α and IL-17A, and at the same time shift immune responses towards the Th2 phenotype and thereby increase the production of anti-inflammatory cytokines, such as IL-4, Il-5 and IL-13 [28]. A recent study in collagen-induced experimental arthritis in mice knee joints [29] could shed some light on this parallel increase of the two opposing cytokines. The authors showed that as IL-17E increased, the production of IL-17A increased at first before decreasing and suggested that a negative regulation exists between the two cytokines with IL-17E slowing down the arthritic inflammation by suppressing IL-17A and by increasing the anti-inflammatory IL-4 [29]. A similar pattern was seen in the current study showing IL-17E to reach its peak at 2 months followed by IL-17A peaking at 4 months before both cytokines retreated. This could suggest that IL-17E has a beneficial role in Charcot patients by increasing the expression of Th2-derived anti-inflammatory cytokines [28] and at the same time counter-balancing the negative effects of IL-17A on the skeleton [29]. In a recent study investigating the role of the potent bone regulating Wnt/β-catenin pathway in Charcot arthropathy patients [30], we were able to show that 4 months after the start of offloading treatment, a significant increase occurred in systemic levels of the Wnt antagonist, dickkopf-1 (Dkk-1), and the Wnt agonist, Wnt-1, both being part of bone remodeling [31, 32]. The present findings showing that the increase in IL-17A and IL-17E following offloading coincided with Dkk-1 and Wnt-1, could suggest that the two systems interact in the control of bone remodeling activity at the early stage of bone repair in Charcot patients. A positive inter-regulatory link between the two systems was recently confirmed in a patient study on fragility fractures showing that a decreased expression of sclerostin, an endogenous inhibitor of the Wnt/β-catenin pathway, was paralleled by a decrease in proinflammatory cytokine expression [33] \thus lending support to a recent suggestion made by Daoussis et al. [34, 35] that mediators of these two systems are critical in joint remodeling and may be appropriate targets in the treatment of bone and joint abnormalities.

As opposed to IL-17A, which did not differ between the three groups at 2 years, IL-17E was significantly higher in Charcot patients versus diabetic controls and healthy (Fig. 2). The latter findings, which signify a continued anti-osteoclatogenic activity in the chronic phase of the disease, may appear surprising considering that Charcot patients showed complete mineralization of the damaged skeleton on radiographs and no presence of active inflammation on MRI (Table 2). However, given the fact that chronic Charcot often is characterized by arch collapse and other bone deformities leading to unnatural high pressure on areas of the foot, there could be a continued need for bone repair and remodeling after patients had resumed full weight-bearing [36]. Thus, an elevation of a cytokine being part of the mechanisms combating osteoclastogenesis may constitute part of a defensive mechanism to preserve the bone in the aftermath of Charcot arthropathy.

Contrary to the rapid increase of systemic IL-17A and IL-17E following offloading treatment, the increase of IL-17F was more gradual and extended over the entire observation period before reaching a level similar to diabetic and healthy controls at 2 years postinclusion (Fig. 3). The specific role of IL-17F on bone has only recently begun to emerge showing this pro-inflammatory cytokine to enable osteoblast maturation and activation which allows bone synthesis to occur as part of its osteoinductive role [37]. Our results showing IL-17F to be suppressed in patients exerting full weight-bearing on the insensate foot at Charcot presentation could suggest that the osteoblast promoting activity of IL-17F was inhibited by excessive motion at the site of injury due to shear loading and that it was gradually restored after the fractured foot had been stabilized by total cast and offloading treatment thereby improving the biomechanical environment for repair (Fig. 2).

## Conclusions

While there is some evidence in support of Th1/Th2 cytokines in the pathophysiology of Charcot arthropathy, this is the first study to support a role for the IL-17 family of proinflammatory cytokines in the pathophysiology of Charcot arthropathy patients. With increased understanding of the bone regulatory mechanisms controlled by members of the IL-17 family, our results showing systemic increase of all three cytokines suggest that they take part in the bone repair and remodeling activity during the recovery phase of Charcot as supported by studies showing direct T-cell involvement in the control of fracture healing [38]. Moreover, the increase of IL-17A and IL-17E within a week after initiation of offloading treatment suggest that activation of these cytokines could have been triggered by fixation and immobilization of the foot as fracture stability is a prerequisite for normal bone repair [38]. Better understanding of the molecular mechanisms triggering and regulating bone repair in of Charcot arthropathy is of particular relevance when considering recent development in the area of drugs targeting the Wnt and IL-17 signaling pathways which hold great promise as a future therapeutic approach to treat common bone disorders [25, 39].
